# Utilising cumulative antibiogram data to enhance antibiotic stewardship capacity in the Cape Coast Teaching Hospital, Ghana

**DOI:** 10.1186/s13756-022-01160-5

**Published:** 2022-10-03

**Authors:** Mavis Puopelle Dakorah, Elizabeth Agyare, Joseph Elikem Efui Acolatse, George Akafity, John Stelling, Victoria J. Chalker, Owen B. Spiller, Nana Benyin Aidoo, Frederick Kumi-Ansah, Daniel Azumah, Stephen Laryea, Robert Incoom, Eric Kofi Ngyedu

**Affiliations:** 1 Cape Coast Teaching Hospital, Cape Coast, Ghana; 2 Microbiology Laboratory, Brigham and Women’s Hospital, Boston, USA; 3 Clinical Services, NHS Blood and Transplant, London, UK; 4 Division of Infection and Immunity, Department of Medical Microbiology, School of Medicine, Cardiff University, Cardiff, UK

**Keywords:** Antimicrobial resistance, Cumulative antibiogram, Antimicrobial stewardship, Quality management, Diagnostic stewardship, Empiric therapy, National standard treatment guidelines

## Abstract

**Background:**

Antimicrobial resistance (AMR) is a major public health challenge with its impact felt disproportionately in Western Sub-Saharan Africa. Routine microbiology investigations serve as a rich source of AMR monitoring and surveillance data. Geographical variations in susceptibility patterns necessitate regional and institutional tracking of resistance patterns to aid in tailored Antimicrobial Stewardship (AMS) interventions to improve antibiotic use in such settings. This study focused on developing a cumulative antibiogram of bacterial isolates from clinical samples at the Cape Coast Teaching Hospital (CCTH). This was ultimately to improve AMS by guiding empiric therapy.

**Methods:**

A hospital-based longitudinal study involving standard microbiological procedures was conducted from 1st January to 31st December 2020. Isolates from routine diagnostic aerobic cultures were identified by colony morphology, Gram staining, and conventional biochemical tests. Isolates were subjected to antibiotic susceptibility testing using Kirby-Bauer disc diffusion. Inhibitory zone diameters were interpreted per the Clinical and Laboratory Standards Institute guidelines and were entered and analysed on the WHONET software using the “first isolate only” principle.

**Results:**

Overall, low to moderate susceptibility was observed in most pathogen-antibiotic combinations analysed in the study. Amikacin showed the highest susceptibility (86%, n = 537/626) against all Gram-negatives with ampicillin exhibiting the lowest (6%, n = 27/480). Among the Gram-positives, the highest susceptibilities were exhibited by gentamicin (78%, n = 124/159), with clindamycin having the lowest susceptibility (27%, n = 41/154). Among the Gram-negatives, 66% (n = 426/648) of the isolates were identified phenotypically as potential extended-spectrum beta-lactamase producers. Multiple multidrug-resistant isolates were also identified among both Gram-positive and Gram-negative isolates. Low to moderate susceptibility was found against first- and second-line antibiotics recommended in the National standard treatment guidelines (NSTG). Laboratory quality management deficiencies and a turnaround time of 3.4 days were the major AMS barriers identified.

**Conclusions:**

Low to moderate susceptibilities coupled with high rates of phenotypic resistance warrant tailoring NSTGs to fit local contexts within CCTH even after considering the biases in these results. The cumulative antibiogram proved a key AMS programme component after its communication to clinicians and subsequent monitoring of its influence on prescribing indicators. This should be adopted to enhance such programmes across the country.

**Supplementary Information:**

The online version contains supplementary material available at 10.1186/s13756-022-01160-5.

## Background

Bacterial antimicrobial resistance (AMR) presents a huge threat to the effective clinical management of bacterial infections with dire global health consequences. Despite increased attention and some improvement in infection prevention and control (IPC) practices which were further highlighted due to the COVID-19 pandemic, there remains a high burden of bacterial infections with alarming rates of AMR leading to morbidity and mortality among patients, especially in low- and middle-income countries (LMICs). A landmark study assessed the global burden of bacterial AMR in 2019 by predictive statistical modelling and estimated that 1.27 million deaths globally were directly attributable to drug-resistant infections annually, while this total could expand to 4.95 million deaths annually if the definition is expanded to include deaths associated with AMR, and not only those attributable to AMR [1]. The Western Sub-Saharan Africa region has the highest death rate for all age groups with 27 deaths per 100,000 directly attributable to AMR and 114.8 deaths per 100,000 more generally associated with AMR [1]. By the year 2050, this figure has been projected to be over 10 million lives lost globally due to AMR and at a cost of $100 trillion in the absence of effective action [2]. The emergence of resistant microorganisms can occur naturally irrespective of the presence of antibacterial agents. However, there is evidence that the inappropriate use of antimicrobials remains the driving force in AMR, providing the necessary selective pressure for the emergence and spread of resistant pathogens. In healthcare settings, the spread of these emergent drug-resistant (DR) organisms limits antimicrobial treatment options leading to prolonged hospital stays, increased expenditure, poor prognosis, and higher mortality rates which further emphasize the need to preserve existing antibiotics for improved patient outcomes and health system benefits [1]. Antimicrobial Stewardship (AMS), defined as a coherent set of actions to promote the responsible use of antimicrobials, is a key pillar regarding health systems strengthening and is crucial for mitigating the effects of AMR in healthcare institutions [3]. AMS in Africa is fairly nascent with publications on the implementation of interventions to effectively reduce excess use of antibiotics while maintaining prompt access to patients who need them in healthcare settings across LMICs on the continent [4].

In Ghana, the Ministry of Health in collaboration with other relevant ministries spanning sectors under the One Health approach recognised the need for AMS in healthcare facilities nationwide as highlighted in the National Action Plan (NAP) and the National AMR policy under the fourth strategic objective to optimise the use of antimicrobial agents in humans [5, 6]. Additionally, the Ministry published National standard treatment guidelines (NSTG) in 2017 to guide prescribers’ choices of medication in cases of empiric and definitive therapy [7]. However, the need for facility-specific policies and prescribing guidelines generated from relevant local evidence and fitted to its context is crucial to effectively mitigate AMR [8]. The WHO document on the development of hospital antibiotic policies identifies the use of local antimicrobial resistance profiles as a key guide for these activities. This is due to the multiple roles such data play in informing appropriate measures to curtail the spread of AMR and to help assess the magnitude of resistance in these contexts. Constant surveillance at the local level is required to establish and monitor AMR patterns to aid in the early detection and response to resistant strains. When incorporated into prescribing guidelines, local surveillance aids effective drug selection and improves empiric treatment, especially across hospitals in LMICs where empiric treatment is a common practice [8].

Many AMS efforts in Ghana have been aimed at evaluating prescribing practices using Point Prevalence Surveys (PPS) such as the projects executed in the Commonwealth Partnerships for Antimicrobial Stewardship (CwPAMS) [9–11]. These are effective in identifying key areas for targeted stewardship interventions, as well as improving prescribing practices through feedback approaches. However, facility-level cumulative antibiograms are also a promising tool in assessing the effectiveness of AMS interventions and promoting the judicious use of antimicrobials to support infection prevention efforts. A cumulative antibiogram is defined as the overall antibiotic susceptibility profiles of a bacterial species to different antimicrobial agents used in microbiological investigations [12]. This approach additionally generates evidence for guiding clinicians in cases of empiric therapy where definitive susceptibility results are unavailable. Therefore, utilising robust standardised methods in antibiogram generation enables accurate comparisons among institutions and limits disparities in AMR patterns due to differences in reporting methods. Even in higher-income countries, adherence to given cumulative antibiogram reporting guidelines among community hospitals in the past was found to be low due to the uncertainty of guidelines, and technicalities involved in its creation. Addressing these concerns helps to increase confidence in the reliability and relevance of observed results [13].

There is currently inadequate availability of cumulative antibiogram data for bacterial pathogens to aid specific interventions that will augment AMS implementation and tailor the NSTGs to patient populations within a given demographic in healthcare facilities in Ghana. As part of a broader stewardship programme, this current study determined a year-long cumulative antibiogram for first isolates of aerobic bacteria isolated from patients across different body sites at the Cape Coast Teaching Hospital (CCTH).

## Materials and methods

### Study design

This prospective longitudinal study investigated AMR in bacterial isolates from clinical samples for 1 year from 1st January 2020 to 31st December 2020.

### Study setting

The study took place at the Microbiology Unit of the Laboratory Department of Cape Coast Teaching Hospital (CCTH), Cape Coast, Ghana. The laboratory department provides diagnostic services for regional referral facilities, out-patients, and about 16 in-patient wards in CCTH, with approximately 10,578 patient admissions in 2020. A total of 5735 cultures and antibiotic susceptibility tests (ASTs), with samples from both the community and healthcare setting was performed in the bacteriology laboratory in 2020.

### Inclusion and exclusion criteria

Only culturable bacterial isolates were included. All tests which were categorized as No Pathogen Identified (NPI), No Significant Growth (NSG), and No Bacterial Growth (NBG) were recorded were excluded from the study. Only isolates with a valid patient ID and accompanying demographics matching the laboratory specimen ID with verifiable results uploaded on the electronic health system were included. Only specimens with at least three susceptibility zone sizes to the different antibiotic panels were included. Results that only reported breakpoint categories without accompanying zone sizes were excluded. All fungal pathogens were excluded. Only the first isolate of a given species encountered for a patient irrespective of its source or susceptibility profile was included (i.e. first isolate only) in accordance with guidance from the CLSI M39 document [12].

### Specimen culture and AST

Primary isolates were obtained by aseptically inoculating solid agar plates: Blood agar, Chocolate agar, MacConkey, Salmonella-Shigella agar, and Cystine–Lactose–Electrolyte-Deficient (CLED) agar (HiMedia, India) with clinical specimens from different body sites. Primary cultures were subjected to aerobic incubation (except Chocolate agar cultures with 10–20% CO_2_) for 24 h at a temperature of 35 ± 2 °C. Purity plates were then prepared from all positive primary cultures by sub-culturing to obtain pure isolates. These were then subjected to Gram staining and conventional bacterial identification methods (colony morphology, carbohydrate utilisation, and enzyme production utilised in indole, urease, citrate, and triple sugar iron biochemical media) under aerobic incubation conditions at 35 ± 2 °C for 18–24 h. For AST, the Kirby-Bauer disc diffusion method was performed for all isolates by inoculating 0.5 McFarland standard of bacterial suspension from the pure isolates on 4 mm-thick Mueller Hinton agar plates (HiMedia, India) and incubated for 24 h aerobically at 35 ± 2 °C. The zones of inhibition diameters were measured with a rule to determine the sensitivity or resistance of the isolates to the antibiotic discs using Clinical and Laboratory Standards Institute (CLSI) 2020 breakpoints [14] incorporated in the WHONET software. The choice of antibiotic disc panels depended on the Gram reaction of the isolate and the availability of discs for routine work in the Microbiology Unit. Gram-negative isolates were tested for susceptibility to: AMK = Amikacin (30 μg), GEN = Gentamicin (10 μg), AMP = Ampicillin (10 μg), SAM = Ampicillin–Sulbactam (10/10 μg), TZP = Piperacillin–Tazobactam (100/10 μg), CTX = Cefotaxime (30 μg), CRO = Ceftriaxone (30 μg), CXM = Cefuroxime (30 μg), CIP = Ciprofloxacin (5 μg), LVX = Levofloxacin(5 μg), NOR = Norfloxacin (10 μg), OFX = Ofloxacin (5 μg), CHL = Chloramphenicol (30 μg), NIT = Nitrofurantoin (300 μg), TCY = Tetracycline (30 μg), and SXT = Trimethoprim-Sulfamethoxazole (1.25/23.75 μg). Gram-positive isolates were tested for susceptibility to: AMK = Amikacin (30 μg), GEN = Gentamicin (10 μg), SAM = Ampicillin–Sulbactam (10/10 μg), AZM = Azithromycin (30 μg), ERY = Erythromycin (15 μg), CTX = Cefotaxime (30 μg), CRO = Ceftriaxone (30 μg), CIP = Ciprofloxacin (5 μg), NOR = Norfloxacin (10 μg), LVX = Levofloxacin (5 μg), OFX = Ofloxacin (5 μg), SPX = Sparfloxacin (5 μg), TCY = Tetracycline (30 μg), NIT = Nitrofurantoin (300 μg), SXT = Trimethoprim-Sulfamethoxazole (1.25/23.75 μg), CHL = Chloramphenicol (30 μg), LNZ = Linezolid (30 μg), LM = Lincomycin (2 μg) and ROX = Roxithromycin (15 μg). Three antibiotic discs were unique to urine isolates. These were NOR, NIT, and LEX = Cephalexin (30 μg), with no breakpoints available for LEX. Due to the absence of breakpoints identified for LM, clindamycin (CLI, 2 μg) was selected on WHONET as a surrogate due to their belonging to the same antibiotic class the lincosamides.

### Statistical analysis

Chi-square analysis was used to determine differences among categorical variables of interest in the study using Microsoft Excel 2016. The *p*-values were reported as two-tailed, and values < 0.05 were considered statistically significant. *Escherichia coli* was used to perform inferential statistical analysis to determine significant differences between outpatient and in-patient populations against antibiotics belonging to different classes of concern namely, AMK (belonging to aminoglycosides), CRO, CTX (both third generation cephalosporins), and CIP (a fluoroquinolone). Zone sizes were interpreted based on the “Performance Standards for Antimicrobial Susceptibility” manual by CLSI 2020 [14] and entered into the configured WHONET (version 20.13.14) database for analysis. The data entered for each cultured isolate included patient ID, sex, date of birth, age, age category, date of admission, prior antibiotic therapy, antibiotic treatment, institution, location, department, location type, specimen number, specimen date, specimen type, report date, reason for sample collection, isolate number, organism, and the antibiotic panel appropriate for the isolate. Manual validation of the WHONET software was performed using line listings in Microsoft Excel 2016 by exporting and manually calculating the susceptibility results of 20 isolates and comparing the generated percentages against the output generated by the software as per Appendix C of the CLSI M39-A4 document “Analysis and Presentation of Cumulative Antimicrobial Susceptibility Test Data; Approved Guideline” [12]. An agreement of 100% of all manual results with the WHONET output was considered acceptable. This was in concordance with Sect. 6.7 [12]. This same guideline was used for the creation of the cumulative antibiogram. WHONET data analysis outputs were generated with the following parameters and exported into Microsoft Excel 2016 for further processing:

1. *Analysis type* study = Susceptibility summary, tables—yes, graphs—yes, antibiotics = all antibiotics
2. *Organisms* ALL = All organisms
3. *One per patient* by patient = First isolate only
4. *Isolates* institution: include—Cape Coast Teaching Hospital
5. *Isolates* specimen date—include: start date 1-Jan-2020 end date 31-Dec-2020
6. *Exclude laboratory isolates* specimen type = ‘qc’, ‘la’, ‘ex’, ‘Department = lab’—yes
7. *Exclude screening isolates* specimen type = ‘sc’, ‘mr’, ‘vr’, ‘cd’—yes
8. *Include isolates that satisfy all of the selection criteria* -yes

All other parameters were used in their default settings.

### Cumulative antibiogram creation with compliance to M39A4 standard

The cumulative antibiogram was assessed for compliance with the guidelines according to Sect. 6.7.2 [12] based on the items listed below:

1. Reporting of isolates that are more than 30 in number or appending a footnote if less than 30 isolates were available but were deemed necessary for inclusion.
2. Definitions provided for all abbreviations.
3. Inclusion of only percentage susceptibilities for antimicrobial agents that are appropriate for the species (derived from Table 2 in CLSI M100 Ed30E).
4. Investigating isolates that did not have a 100% susceptibility to antimicrobial agents in the CLSI M100 Ed30E [14] with only “susceptible” interpretive criteria.

Additionally, isolates with intrinsic resistance to certain antibiotic agents were represented with an “R” as per Appendix J [12]. Validated data fulfilling the above criteria were then exported into a Microsoft Word 2016 template and converted into PDF files.

## Ethics statement

Ethical approval was obtained from the Cape Coast Teaching Hospital Research Ethical Review Committee (CCTHERC/EC/2019/075). A waiver of consent was obtained for all diagnostic isolates as this was part of routine microbiological diagnostic procedures in the hospital. All data obtained from the study were concurrently entered and encrypted on WHONET and kept confidential throughout the study. Positive cultures were communicated to requesting physicians for the management of patients as routinely done in the hospital.

## Results

### Sample general characteristics

In total, 829 out of 912 positive cultures from various clinical specimens were included in the study to generate the cumulative antibiogram of respective isolates, which formed the basis of all the analyses. Out of these, urine cultures contributed the largest number of isolates, 363 samples (43.8%), followed by blood cultures with 156 samples (18.8%). Gram stain classification indicated a dominant population of Gram-negative bacteria with 648 isolates (78.2%) while the remaining 181 isolates (21.8%) were Gram-positive organisms. The most common sample was for outpatients and accounted for 379 (45.7%) samples with respect to specimens’ location type. By age category, a high count of positive cultures was observed among specimens from adults (≥ 18 years) with 629 (75.9%) isolates and new-born (< 28 days) specimens contributing the least with 91 (11.0%) isolates. Regarding sex, female patients contributed 522 (63.0%) isolates whereas 307 (37.0%) were from males (Table 1).

**Table 1:** Patient demographics, specimen types, and bacterial isolates distribution

Variables	All positive Cultures		Gram-negative isolates		Gram-positive isolates	
	Number	%	Number	%	Number	%
*Sex*						
Female	522	63.0	426	65.7	96	53.0
Male	307	37.0	222	34.3	85	47.0
*Age category*						
New-born	91	11.0	49	7.6	42	23.2
Paediatrics	109	13.2	75	11.6	34	18.8
Adults	629	75.9	524	80.9	105	58.0
*Location type*						
Emergency unit	49	5.9	35	5.4	14	7.7
Intensive care unit	65	7.8	42	6.5	23	12.7
Inpatient	336	40.5	264	40.7	72	39.8
Outpatient	379	45.7	307	47.4	72	39.8
*Specimen type*						
Blood	156	18.8	68	10.5	88	48.6
Sputum	68	8.2	58	9.0	10	5.5
Urine	363	43.8	350	54.0	13	7.2
Vagina	64	7.7	40	6.2	24	13.3
Wound	134	16.2	107	16.5	27	14.9
Others	44	5.3	25	3.9	19	10.5
Overall total	829	100.0	648	78.2	181	21.8

### Gram-negative cumulative antibiogram

The susceptibility patterns for the 566 Enterobacteriaceae were not far from the general cumulative antibiogram observation among all the Gram-negative isolates. Comparatively, the susceptibility to aminoglycosides was high, ranging from 47 to 87% for GEN and AMK, followed by the fluoroquinolone group (23–52%), penicillins (4–42%), cephalosporins (14–22%), and other antibiotics (16–37%) which included NIT, CHL, and SXT. β-lactams (penicillins and cephalosporins) performed poorly against all Enterobacteriaceae, and AMP particularly showed very low susceptibilities with urine isolates from *Klebsiella* and *Enterobacter spp*. recording no (0%) susceptibility. This was expected due to intrinsic resistance described in *K. pneumoniae, E. cloacae*, and *E. aerogenes spp*. *P. aeruginosa,* on the other hand, demonstrated an intrinsic resistance to β-lactams with or without an inhibitor except for TZP to which 20% (n = 5) of the few isolates tested were susceptible. For all non-fermenters, AMK still exhibited the highest susceptibility (76%) though reduced as compared to Enterobacteriaceae. GEN, AMP, CTX, CIP, and NOR all had marginally higher susceptibilities for all non-fermenters with the largest difference seen in AMP (4% vs 14%) compared with all Enterobacteriaceae. Among *Proteus spp*., OFX (68%) had the second-highest susceptibility after AMK. OFX also had the third-highest susceptibility for *P. aeruginosa* (46%).

Furthermore, susceptibilities to the combination antibiotic agents SAM and TZP differed among species. Susceptibilities to non-urine isolates of *E*. *coli*, *Citrobacter,* and *Enterobacter spp*. against SAM were 0% whereas that of all *Pseudomonas spp.* from non-urine specimens was 50%. For TZP, susceptibilities ranged from 33 to 67% for all tested species from various specimens. In urine isolates, susceptibilities to SAM ranged from 25 to 46%. among all species tested.

Regarding the aminoglycosides AMK and GEN, high susceptibilities were recorded from non-urine specimens for *E. coli* (95% and 60%) and *Citrobacter spp*. (88% and 52%) isolates compared to those from urine specimens. For *Klebsiella spp*., higher susceptibilities were demonstrated for urine specimens (93% and 67%) versus those in non-urine specimens (91% and 38%) for AMK and GEN respectively. Also, isolates of *Enterobacter spp*. from urine specimens were more susceptible to AMK compared to isolates from non-urine specimens but demonstrated a different pattern against GEN with non-urine isolates exhibiting higher susceptibility.

### Gram-positive cumulative antibiogram

Among all 181 Gram-positive isolates, none of the six isolates tested against NIT were resistant (100%) to this antibiotic while the lowest susceptibility was recorded for CLI (27%). Coagulase Negative *Staphylococcus* (CoNS), being the most isolated *Staphylococcus* spp. showed a high degree of susceptibility to NOR (100%), OFX (100%), CRO (100%), and CHL (100%) but low susceptibilities to ERY (36%), CTX (33%), CIP (45%) and CLI (20%). CoNS susceptibility to AMK and GEN was found to be 50% and 73% respectively. In the case of *S. aureus* isolates, higher susceptibility was observed with GEN (90%) and SPX (89%) whilst low susceptibilities of 0%, 21%, 33%, 22%, and 35% were recorded for, NOR, TCY, OFX, AMK, and SXT respectively. Susceptibilities showed all *Staphylococcus spp.* to be highly sensitive to GEN, SPX, and NIT, with all susceptibilities being above 70% regarding these antibiotics. *S. aureus* strains showed higher susceptibilities to GEN (90%) and SPX (89%) compared to other *Staphylococcus spp.* while exhibiting low susceptibilities of 47% and 50% to β- lactams CTX and CRO respectively with SAM showing comparatively higher susceptibility of 61%. LNZ susceptibility was moderate at 62% for all Gram-positive organisms tested.

Summaries of the susceptibility profiles of Gram-negative and Gram-positive bacteria identified at the CCTH bacteriology laboratory against the panel of tested antibiotics are shown in Tables 2 and 3. Both cumulative antibiograms were fully compliant with the standards used.

**Table 2:** Percent susceptible isolates from all sources (N = 648) of gram-negative bacteria from Cape Coast Teaching Hospital

Gram-negative Bacteria	Isolates	Aminoglycosides		ß-lactams						Fluoroquinolones				Other			
				Penicillins			Cephalosporins										
	N	AMK	GEN	AMP	SAM	TZP^a^	CTX	CRO	CXM	CIP	LVX	NOR^b^	OFX	CHL	NIT^c^	TCY	SXT
All Gram-negative organisms	648	86	48	6	42	38	16	22	16	20	31	24	59	36	34	16	17
All Enterobacteriaceae	566	87	47	4	42	39	14	22	17	19	32	23	52	37	37	16	18
All *Escherichia coli spp.*	195	87	48	5	44	50	13	21	17	16	28	26	44	42	47	13	16
*Escherichia coli* (urine)	144	85	40	2	46	–	5	19	11	12	25	26	39	42	47	11	15
*Escherichia coli* (non-urine)^d^	57	95	60	7	0^e^	50	25	25	26	26	36	–	55	43	–	18	18
All *Klebsiella spp.*	74	93	43	6	46	33	24	29	21	25	43	30	62	36	18	30	25
*Klebsiella sp*. (urine)	41	93	67	0	46	–	17	28	17	19	43	30	59	33	18	34	26
*Klebsiella sp*. (non-urine)^d^	35	91	38	7	43^e^	33	25	28	21	31	44	–	65	37	–	24	24
All *Citrobacter spp.*	144	84	45	3	21^e^	67	12	20	17	18	30	7	52	31	20	14	19
*Citrobacter sp.* (urine)	69	81	36	2	25^e^	–	12	12	15	12	16	7	32	22	20	7	15
Citrobacter sp. (non-urine)^d^	78	88	52	4	0^e^	67	13	27	18	24	41	–	69	38	–	20	23
All *Enterobacter spp*.	78	91	42	2	43^e^	0	8	18	7	20	32	31	52	34	62	18	11
*Enterobacter sp.* (urine)	49	92	42	0	46^e^	–	7	22	9	15	26	31	43	35	62	15	11
*Enterobacter sp*. (non-urine)^d,e^	29	90	43	4	0^e^	0	10	10	4	28	42	–	69	33	–	24	10
All non-fermenting Gram-negative rods	81	76	57	14	40^e^	38	22	21	11	25	22	33	47	30	0	11	10
All *Pseudomonas spp.*	70	77	51	2	36^e^	29	10	11	4	19	15	33	42	20	0	10	11
All *Pseudomonas spp.* (non-urine)^e^	46	76	50	3	50^e^	33	12	14	3	23	16	–	40	24	–	7	11
*Pseudomonas aeruginosa*	35	86	56	R	R	20	R	R	R	20	18	29	46	R	0	R	R
*Pseudomonas sp*. (non-aeruginosa)	35	67	47	3	33^e^	50	14	21	3	18	12	50	38	28	0	9	11
All *Proteus spp.*	55	83	53	8	43^e^	0	27	29	28	26	37	0	68	35	33	9	24
*Proteus sp*. (non-urine)^d^	39	79	57	11	100^e^	0	34	35	29	32	41	–	75	37	–	13	26

%S = Percent of isolates susceptible, all organisms were tested by disc diffusion, N = Number, *spp*. = species, R = intrinsically resistant, (‒) = No data available, small number of isolates tested (N < 30), antimicrobial agent is not indicated, or not effective clinically. Interpretation standard: CLSI M100 ED30:2020. Presentation standard: CLSI M39-A4:2014. Data analysis: WHONET 2020 (20.13.14)

Data source: Cape Coast Teaching Hospital Routine Antimicrobial Surveillance. Data shown is from surveillance of routine diagnostic cultures performed in the institution from 1st January to 31st December 2020. Version 1.1 (12/02/2021)

AMK, Amikacin; GEN, Gentamicin; AMP, Ampicillin; SAM, Ampicillin–sulbactam; TZP, Piperacillin–tazobactam; CTX, Cefotaxime; CRO, Ceftriaxone; CXM, Cefuroxime; CIP, Ciprofloxacin; LVX, Levofloxacin; NOR, Norfloxacin; OFX, Ofloxacin; CHL, Chloramphenicol; NIT, Nitrofurantoin; TCY, Tetracycline; and SXT, Trimethoprim–sulfamethoxazole

The percent of isolates susceptible (%S) for each organism/antimicrobial combination was generated by including the first isolate only of that organism encountered on a given patient during 2020 (de-duplicated data)

^a^TZP: Piperacillin–Tazobactam few isolates were tested (N < 30) and the percentage susceptible should be interpreted with caution

^b^NOR: Norfloxacin data from urine isolates only

^c^NIT: Nitrofurantoin data from urine isolates only

^d^Isolates were tested against antibiotics in urine panel only (NOR & NIT)

^e^A small number of isolates were tested (N < 30), and the percentage susceptible should be interpreted with caution

**Table 3:** Percent susceptible isolates of all sources (N = 181) of gram-positive bacteria Cape Coast Teaching Hospital

Organism	Isolates	Aminoglycosides		Penicillin	Macrolides		Cephalosporins		Fluoroquinolones					Other					
	N	AMK^a^	GEN	SAM	AZM^b^	ERY^c^	CTX	CRO^d^	CIP	NOR^e^	LVX	OFX^f^	SPX^g^	TCY	NIT^h^	SXT	CHL^i^	LNZ	CLI^j^
All Gram-positive organisms	181	44	78	59	64	39	37	50	46	33	55	50	85	29	100	30	67	62	27
*Staphylococcus sp.*	166	35	78	58	64	39	36	75	48	33	54	33	85	31	100	31	67	62	26
*Staphylococcus aureus ss. aureus*	45	22	90	61	67	43	47	50	60	0	58	33	89	21	100	35	50	58	44
*Staphylococcus,* coagulase negative	113	50	73	61	67	36	33	100	45	100	55	100	80	35	100	31	100	66	20
*Staphylococcus*, coagulase negative(blood)	70	20	74	67	63	50	35	100	52	–	56	–	88	37	–	35	–	79	27

AMK, Amikacin; GEN, Gentamicin; SAM, Ampicillin–sulbactam; AZM, Azithromycin; ERY, Erythromycin; CTX, Cefotaxime; CRO, Ceftriaxone; CIP, Ciprofloxacin; NOR, Norfloxacin; LVX, Levofloxacin; OFX, Ofloxacin; SPX, Sparfloxacin; TCY, Tetracycline; NIT, Nitrofurantoin; SXT, Trimethoprim–sulfamethoxazole; CHL, Chloramphenicol; LNZ, Linezolid; CLI, Clindamycin; and N, total number of isolates

The percent of isolates susceptible (%S) for each organism/antimicrobial combination was generated by including the first isolate only of that organism encountered on a given patient during 2020 (de-duplicated data)

^e^NOR: Norfloxacin data from urine isolates only

^h^NIT: Nitrofurantoin data from urine isolates only

^j^CLI: Clindamycin breakpoints were used as a surrogate for Lincomycin breakpoints during susceptibility reporting

^a,b,c,d,f,g,i,j^AMK: Amikacin; AZM: Azithromycin; ERY: Erythromycin; CRO: Ceftriaxone; OFX: Ofloxacin; SPX: Sparfloxacin; CHL: Chloramphenicol—less than 30 isolates (N < 30) were tested against these antibiotics hence results should be interpreted with caution

%S = Percent of isolates susceptible, all organisms were tested by disc diffusion, N = Number, *spp*. = species, R = intrinsically resistant, (‒) = No data available, small number of isolates tested (N < 30), antimicrobial agent is not indicated, or not effective clinically. Interpretation standard: CLSI M100 ED30:2020. Presentation standard: CLSI M39-A4:2014. Data analysis: WHONET 2020 (20.13.14)

Data source: Cape Coast Teaching Hospital Routine Antimicrobial Surveillance. Data shown is from surveillance of routine diagnostic cultures performed in the institution from 1st January to 31st December 2020. Version 1.1 (12/02/2021)

Chi-square statistical analysis showed no significant differences among the pathogen-antibiotic combinations of CTX (*p* = 0.87), CRO (*p* = 0.97), AMK (*p* = 0.50), and CIP (*p* = 0.94) against *E. coli*. Additionally, for antibiotic-pathogen susceptibilities appended with footnotes whereby small numbers of isolates were tested, full data on the total number of such isolates are available in Additional file: 1 and 2 for Gram-negative and Gram-positive cumulative antibiograms respectively.

### WHONET priority isolate alerts

WHONET offers a list of approximately 190 pre-defined isolate-level microbiology alerts for “important species”, “important resistance”, and “quality control” categorized as “high”, “medium”, and “low” priority. A total of 14 “high” and “medium” priority alert rules were raised by the WHONET software. For Enterobacteriaceae, 438 isolates were suspected to be ESBL-producers based on their phenotypes from susceptibility testing in concordance with results from the cumulative antibiogram with an additional alert for infection control regarding these isolates. This was followed by 70 isolates from this same family being AMK non-susceptible as an “important resistance” of medium priority.

Five medium-priority quality control (QC) alert rules were triggered. Discordant results for 70 Enterobacteriaceae isolates and one *P. aeruginosa* isolate were identified to the aminoglycoside antibiotics AMK and GEN. These were manifested phenotypically as GEN susceptible and AMK resistant isolates. Discordant results for cephems were also observed among 34 Enterobacteriaceae isolates with the fourth QC alert belonging to two *Proteus spp.* which were NIT susceptible. Finally, a QC alert was identified based on the wrong testing method, namely; disc-diffusion testing for *S. pneumoniae* against ß-lactam antibiotics.

For high priority alerts, five alerts involving *Streptococcus, Staphylococcus, and Salmonella spp.* were identified. For *Streptococcus spp*. one third-generation cephalosporin and four linezolid non-susceptible isolates were identified. An additional 46 isolates belonging to *Staphylococcus spp.* were also identified to be LNZ non-susceptible. The remaining alerts were attributed to one *Salmonella* isolate which was both third generation cephalosporin and fluoroquinolone non-susceptible. Six of these alerts were suggested for saving and subsequent sending to a reference lab for further testing as well as implementing infection control measures as indicated by WHONET (Table 4).

**Table 4:** WHONET isolate alerts summary

Rule number	Organisms	Alert	Number of isolates	Priority	Quality control	Important species	Important resistance	Infection control
23	Enterobacteriaceae	Amikacin = Non-susceptible	70	Medium	−	−	+	−
24	Enterobacteriaceae	Aminoglycosides = Discordant results	11	Medium	+	−	−	−
26	Enterobacteriaceae	Cephems = Discordant results	34	Medium	+	−	−	−
30	Enterobacteriaceae	Possible ESBL-producing Enterobacteriaceae	438	Medium	−	−	+	+
71	*Proteus sp.*	Nitrofurantoin = Susceptible	2	Medium	+	−	−	−
78	*Pseudomonas aeruginosa*	Aminoglycosides = Discordant results	1	Medium	+	−	−	−
85	*Salmonella sp.*	Cephalosporin III = Non-susceptible	1	High	−	−	+	+
87	*Salmonella sp.*	Fluoroquinolones = Non-susceptible	1	High	−	−	+	+
88	*Salmonella sp.*	Important species	1	Medium	−	+	−	+
101	*Staphylococcus sp.*	Linezolid = Non-susceptible	46	High	−	−	+	+
109	*Streptococcus pneumoniae*	Beta-lactams = Tested by disc diffusion	3	Medium	+	−	−	−
116	*Streptococcus sp.*	Linezolid = Non-susceptible	4	High	−	−	+	+
119	*Streptococcus viridans*	Penicillin or Ampicillin = Non-susceptible	1	Medium	−	−	+	−
121	*Streptococcus,* beta-haemolytic	Cephalosporin III = Non-susceptible	1	High	−	−	+	+

“ + ” represents a positive alert whereas “ − “ represents no alert for each rule

## Discussion

This study served as the baseline for informing local empiric prescribing guidelines by aiding in developing a prescribing protocol, a facility-level AMS policy, and making improvements from implementing this AMS tool in a Ghanaian tertiary hospital. Quality improvements included upskilling the local staff to create a cumulative antibiogram and meet all requirements needed per the guidance documentation. These included updating laboratory request forms to include antibiotic use and addition of data to the e-health system, enabling the production of a cumulative antibiogram, and evaluating local staff’s compliance with the requirements stated in the guidance documentation. Cumulative antibiograms are valuable tools for clinical decision support and epidemiological surveillance of emerging resistance trends. They can also be leveraged in AMS programmes in hospital settings by guiding empiric therapy [12]. In this study, two cumulative antibiograms were developed based on the classification of bacterial isolates according to their Gram stain reactions (Gram-positive and Gram-negative) due to the differences in organism-antibiotic combinations used in testing for these two groups. The cumulative antibiograms were not further subdivided according to inpatient and outpatient departments despite the knowledge of possible differences in susceptibility patterns due to hospital- (HAI) versus community-acquired infections (CAI) [15]. Despite a larger volume of patients seen in the outpatient setting the sum of inpatient, emergency, and intensive care units’ specimens which usually have patients with greater pathology (and a greater risk that the infections may represent HAIs) accounted for 450 isolates as compared to the outpatient unit with 379 isolates (Table 1). Additionally, no statistically significant differences were found between inpatient and outpatient susceptibility patterns using *E. coli* which is considered a good indicator for AMR surveillance due to its wider implication in many disease conditions and its ease of isolation and culture [16]. However, no adjustments were made for the multiple comparisons (Additional file 5).

Regarding ß–lactams, *E. coli* showed very low susceptibility to AMP (5%) but saw an appreciable increase to 44% and 50% with combinations including ß-lactamase inhibitors namely; SAM and TZP respectively. This may indicate the presence of Ambler Class A ESBLs which show increased susceptibility to clavulanic acid [15]. Knowledge of co-harbouring of multiple resistance genes within this species both in Ghana [17–20] and other African countries may explain the overall low susceptibility to these drugs. Among cephalosporins, all three drugs tested showed low susceptibility percentages among *E. coli*. CRO had the highest susceptibility of the three with 21% which acts as a surrogate to determine ESBL-producing isolates. These cumulative antibiogram results corroborate the WHONET alerts indicating the presence of possible ESBL-producers. This is a cause for concern within the context of CCTH and the country at large due to CRO being classified as a “Watch” antibiotic according to the WHO Access, Watch, and Reserve (AWaRe) classification based on their importance and relatively high risk of selection for bacterial resistance [21]. Cephalosporins are also suggested as empiric first and second-line treatments in the NSTGs for many conditions in which *E. coli* may be implicated which may lead to treatment failure if sensitivity testing is not carried out promptly due to low susceptibility from the study’s findings. Biases may exist especially with CCTH status as a tertiary referral hospital with patients attending this hospital possibly encountering initial treatment failures, having a recent hospitalisation history, and having complicated medical presentations which would lead to lower susceptibility rates observed within the institution than in the general community or lower-level hospitals in the country. Clinician sampling practices may also influence the observed susceptibilities as patients who respond to empiric therapy may not have specimens taken for analysis at the lab, especially in outpatient settings [12]. Other top-priority Gram-negative bacteria include; *Klebsiella spp.* and *Enterobacter spp.* defined by WHO as part of the ESKAPE pathogens (*Enterococcus faecium, Staphylococcus aureus, K. pneumoniae, Acinetobacter baumannii, P. aeruginosa, Enterobacter spp.*) [22] also exhibited similar patterns with 29% and 18% susceptibility to CRO respectively and increased susceptibility to SAM and TZP. Additionally, frequent use of ß-lactam antibiotics used in CCTH (unpublished data) and other health facilities across the country especially the third-generation cephalosporins [23–26] may further exacerbate this problem. *S. aureus* recorded susceptibilities of below 51% to CRO and CTX but saw an increase to SAM (61%). Though these antibiotics are no longer recommended for testing against *S. aureus*, the non-availability of antibiotic panels which include cefoxitin and oxacillin limit resistance mechanism inferences for such isolates and warrant further investigation to ascertain if these are Methicillin-Resistant *Staphylococcus aureus* (MRSA) isolates as these have been reported in other settings in Ghana [27–29].

OFX had the highest susceptibility among the fluoroquinolones tested against Enterobacteriaceae with higher susceptibilities achieved in non-urine isolates for *E. coli* (55%)*, Klebsiella spp.* (65%)*, Citrobacter spp.* (69%), and *Enterobacter spp.* (69%) as compared to urine isolates of the same species with susceptibilities of 39%, 59%, 32%, and 43% respectively. This is also alarming due to the use of fluoroquinolones in the treatment of urinary tract infections (UTIs) and sometimes gonorrhoea especially given its oral option for outpatient use. Another option for UTIs in the form of NIT showed poor susceptibility of < 50% to all Gram-negatives tested except *Enterobacter spp.* (62%) which does not make it a feasible alternative for the low susceptibilities observed within the fluoroquinolone class. Despite small sample sizes and some differences in methodology used, reports from other studies in Ghana indicate the growing problem of fluoroquinolone resistance, especially in *E. coli* which may result in treatment failure if used empirically within such settings [18, 30–32]. This pathogen drug-combination was responsible for approximately 50,000–100,000 AMR-attributable deaths globally in 2019. While in Ghana, *E. coli* resistance to fluoroquinolones was estimated to range from 30% to just below 40% [1]. Additionally, one *Salmonella* isolate recovered during this period reported fluoroquinolone non-susceptibility which is of high priority for both IPC and surveillance considering limited therapeutic options. This isolate was however not stored for further examination at the National Reference Laboratory (NRL) due to inadequate resources. Increasing fluoroquinolone resistance has been identified among gastrointestinal pathogens especially *Salmonella spp.* from studies across Ghana and globally [33–35] and future monitoring and iterations of an institutional cumulative antibiogram will be needed to determine the magnitude of this resistance pattern.

The aminoglycosides GEN and AMK had variable levels of susceptibility from moderate to high between Gram-negative and Gram-positive bacterial isolates. Among the Gram-negatives, high susceptibilities to AMK (77–93%) were observed with *Pseudomonas spp*. having the lowest, whereas for GEN, low to moderate susceptibilities were seen among these isolates ranging from 42 to 53%. Higher susceptibilities in both *S. aureus* (90%) and Coagulase Negative *Staphylococcus* (CoNS) (73%) were observed against GEN. Lower AMK susceptibility patterns may have been due to the use of general breakpoint interpretations per WHONET default settings which are no longer recommended and lab quality management deficiencies as AMK is known to achieve equivalent efficacy in lower doses for UTIs compared with GEN [36] and was flagged by the WHONET software as requiring QC. Future iterations of the cumulative antibiogram will exclude this drug-organism combination unless results can be confirmed by other more sensitive and specific methods.

A noteworthy observation from the Gram-positive cumulative antibiogram was the susceptibility of *Staphylococci spp.* to LNZ. Despite this antibiotic’s unavailability in Ghana, moderate susceptibility of 58% was observed among *S. aureus* species cultured throughout the year. LNZ remains one of the most potent antibiotics against *Staphylococcus spp.* infections worldwide and is a suitable therapeutic option for infections caused by both MRSA and Vancomycin-Resistant *Staphylococcus aureus* (VRSA). The option of LNZ use within CCTH in future must be subject to proper quality assurance and testing. This use must also be based on disease severity and clinical scenario as the true susceptibility to this antibiotic is likely to be higher than that found in our study. In this case, false resistance among different test methods has been observed [37] which could have been the case here and none of the isolates were sent to the NRL for retesting and confirmation. Further studies are needed to determine the true extent of susceptibilities among Gram-positives to this drug.

Multidrug resistance (MDR) was defined according to Magiorakos et al*.* [38] for *S. aureus* and Enterobacteriaceae as non-susceptible to ≥ 1 agent in ≥ 3 selected antimicrobial categories [38]. Nine *S. aureus* isolates were found to be MDR according to the above-stated definition with many of the isolates being non-susceptible to TCY, CIP, SXT, CLI, and each isolate being tested against at least three antibacterial classes (Additional file 3). These isolates were from both inpatient and outpatient settings in the facility. MDR was more common among the Gram-negatives with 426 isolates identified with the most common resistance profiles being non-susceptibility to CTX, CRO, CXM, CIP, CHL, and AMP which is reflected in the cumulative antibiogram (Additional file 4). This definition excluded intrinsic resistance to certain classes of antimicrobial agents specified leading to an overall fewer number of MDR isolates than originally reported by WHONET. For most *Pseudomonas spp.* isolates within the database (n = 20), insufficient data due to limited classes of antibiotics with activity against these organisms tested against them prevented their MDR classification. Updated panels that include more antipseudomonal drugs could be utilised to determine their MDR status in the future.

*Pseudomonas* *aeruginosa* had a low susceptibility to the antipseudomonal penicillin + ß-lactamase inhibitor TZP of 20% however, only a few isolates (n = 5) were tested against this drug due to a change in antibiotic discs during the study. Moderate levels of susceptibility to GEN (56%) and OFX (46%) were observed with AMK having the highest susceptibility among *P. aeruginosa* isolates in CCTH at 86%. Some international guidelines recommend the use of aminoglycosides for infections both within and outside the urinary tract [39] and these local susceptibility data support AMK as the most appropriate choice given the unavailability of newer agents such ceftazidime-avibactam or ceftolozane-tazobactam in this setting.

SXT also showed low susceptibility across all organisms in both Gram-positive (30%) and Gram-negative cumulative antibiograms (17%). This antibiotic is routinely used in HIV-positive patients to prevent opportunistic infections in Ghana. These findings warrant further studies to assess this drug's utility in this patient demographic and its efficacy in such cases. These results will act as the primer for a review of the current treatment guidelines as a future follow-up study to generate local recommendations adapted to the institution for conditions covered under the scope of the samples and isolates analysed in this study.

### Diagnostic stewardship

Despite the existence of national guidelines, prescribing is usually down to individual clinical preference and expertise. The data showed 298 inpatients out of a total of 450 (66.2%), including emergency and intensive care units, whose samples were submitted to the microbiology laboratory for testing were already started empirically on at least one antibiotic with an average of 1.7 (95% C.I. 1.5 to 2.0) antibiotics per patient. This was similar to findings of a PPS conducted in December of 2020 in the institution conducted conjointly with this as a part of the entire AMS programme which showed that an average of 2.3 (95% C.I. 2.0 to 2.6) antibiotics were given to patients during their stay in the institution after a preliminary analysis which is to be published elsewhere. This occurrence could be attributed to several reasons but most importantly for our study was the turnaround time (TAT) for results to reach prescribers. The average time taken in days for reports to be uploaded to the electronic health system from sample reception was 3.4 days which could be a possible reason for empiric treatment by prescribers before or pending culture results given the high burden of bacterial infections in West Africa as a whole. Further AMS interventions will probe into definitive therapy for such patients to assess the influence of culture results on guiding or changing therapy.

Quality Management System deficiencies were also identified from alerts that are needed to optimise patient care in healthcare settings, especially in LMICs where resources for this are limited [40–42]. The prospective use of WHONET could prove to be valuable in certain Quality System Essentials (QSEs) like Information Management, Documents and Records, and Process Control [43]. This will ultimately improve the laboratory’s output with concomitant benefits for patient outcomes and data provision for surveillance.

All these findings were used in concert with PPS data as part of a wider study to develop the institution’s first-ever draft AMS local policy and to begin a critical assessment for adaptation of the NSTGs based on local susceptibility for empiric treatment in the facility after permission was sought from the Ministry of Health showing its feasibility of implementation and benefits in such a setting.

### Limitations of the study

The significant limitation of this study was the reduction in sample size due to COVID-19 and some quality management deficiencies warranting the exclusion of isolates. Secondly, the use of manual methods of identification, AST, and the antibiotic discs available for this limited the inclusion of some relevant pathogen-antibiotic combinations used in clinical practice like cefoxitin, while including others of limited relevance (i.e. SAM, CRO, CTX), especially for *Staphylococci* isolates. Additionally, a limited number of isolates for certain bacteria species hindered the study. According to CLSI recommendations, a minimum of 30 isolates are needed to maintain statistical validity for cumulative antibiogram reporting and therefore suggests adding the previous year’s data if 30 isolates are not reached and the percentage susceptibilities have not changed significantly, however, poor data archiving in the laboratory unit made it impossible to retrieve such data. Many specimens received at the laboratory were from patients already on antibiotic therapy, this may bias the results to exclude isolates sensitive to the antibiotics and lead to a negative test result as well as over-representing resistant isolates due to the administered therapy before the specimens were taken. Biases in culturing practices such as the sampling of patients with prolonged medical histories or treatment failures who were treated solely empirically may have also led to observing lower susceptibilities than is true of the patient population within CCTH. This study is not ideally designed to monitor patients receiving multiple antibiotics with complex and multi-factorial infections caused by differing bacterial species. This could drive the emergence of resistance among implicit pathogens and further exaggerate the true resistance among first isolates. Despite LM and CLI belonging to the same antibiotic class, there is no guarantee that they have the same breakpoints due to differences in molecular weight or ionic characteristics, which could impact diffusion rates. Finally, the inability to perform anaerobic cultures, to send samples that had an alert to a reference laboratory, and the inability to implement IPC measures due to results analysis lagging behind the management of the patient limited the study and additional benefits of this AMS tool.

## Conclusions

This study addressed a gap in understanding the extent of AMR by providing baseline cumulative antibiogram data of clinical isolates in a Ghanaian tertiary hospital. The generated cumulative antibiogram will serve as an important tool in combating AMR through improved prescribing practices at all levels of healthcare delivery. The high levels of resistance among the Gram-negative and Gram-positive isolates found in the study underline the need for reliable AST data through routine cultures that can contribute to consistent and regular cumulative antibiogram development and dissemination to inform appropriate antibiotic use. These findings also confirm the need for AMR monitoring and surveillance at both private and government facilities, to establish the local prevalence and aid an effective AMS programme. Cumulative antibiograms could serve as a tool for enhancing the capacity in LMIC healthcare institutions capable of providing microbiological services to generate much-needed data on local resistance patterns to inform treatment guidelines and policy, as well as provide support to other less-equipped facilities which lack this through pooled or regional cumulative antibiogram development. The current data also highlights the need for further assessment and tailoring of the current recommended empiric first- and second-line therapies embedded within the NSTGs to the contexts of healthcare facilities based on AMR patterns observed in their locality. These efforts fall in line with strategic objectives 6.1.1.1 and 6.2.1.1 of the NAP [6] to contribute to establishing national monitoring systems of antimicrobial use and AMR surveillance to inform policy and improve the quality of laboratory diagnostic services to inform the selection and prescribing of antibiotics.

## Supplementary Information

**Additional file 1**. Gram-negative Cumulative Antibiogram Raw Data. Total number of Gram-negative isolates tested against each antibiotic to produce cumulative antibiogram percentage susceptibilities.

**Additional file 2.** Gram-positive Cumulative Antibiogram Raw Data. Total number of Gram-positive isolates tested against each antibiotic to produce cumulative antibiogram percentage susceptibilities.

**Additional file 3.** Multidrug-Resistant S. aureus isolates raw data. Total number of S. aureus isolates classified as multidrug-resistant together with the number of classes of antibiotics they were tested against and zone diameters against antibiotics tested.

**Additional file 4.** Gram-negative multidrug-resistant isolates raw data. Total number of Gram-negative isolates classified as multidrug-resistant together with the number of classes of antibiotics they were tested against and zone diameters against antibiotics tested.

**Additional file 5.** Inpatient and outpatient raw data for Chi-square analysis. Raw inpatient and outpatient data used in E. coli Chi-square statistical analysis against amikacin, ceftriaxone, cefotaxime, and ciprofloxacin.

**Additional file 6.** Deidentified complete raw dataset. Raw dataset including duplicate isolates not subjected to the “first isolate only” rule collected during the study with all patient identifiers removed.

## Data Availability

De-identified data without any demographic data (i.e. patient ID, sex, date of birth, age, date of admission, institution, location, department, and specimen ID) is available in Additional file 6. The entire dataset with encrypted IDs can be made available from the authors upon reasonable request and in agreement with the management policy at the Cape Coast Teaching Hospital.

## Abbreviations

AMK: Amikacin
AMP: Ampicillin
AMR: Antimicrobial resistance
AMS: Antimicrobial stewardship
AST: Antimicrobial susceptibility testing
AZM: Azithromycin
CAI: Community-acquired infection
CCTH: Cape Coast Teaching Hospital
CHL: Chloramphenicol
CIP: Ciprofloxacin
CLED: Cystine–lactose–electrolyte-deficient
CLI: Clindamycin
CLSI: Clinical and Laboratory Standards Institute
COVID-19: Coronavirus disease 2019
CRO: Ceftriaxone
CTX: Cefotaxime
CXM: Cefuroxime
ERY: Erythromycin
ESBL: Extended-spectrum beta-lactamase
ESKAPE: *Enterococcus faecium, Staphylococcus aureus, K. pneumoniae, Acinetobacter baumannii, P. aeruginosa, Enterobacter* Spp.
GEN: Gentamicin
HAI: Hospital-acquired infection
HIV: Human immunodeficiency virus
IDSA: Infectious Diseases Society of America
IPC: Infection prevention and control
LEX: Cephalexin
LM: Lincomycin
LMIC: Low–middle-income country
LNZ: Linezolid
LVX: Levofloxacin
MDR: Multidrug resistant
MRSA: Methicillin-resistant *Staphylococcus aureus*
NAP: National action plan
NBG: No bacterial growth
NIT: Nitrofurantoin
NOR: Norfloxacin
NPI: No pathogen identified
NRL: National Reference Laboratory
NSG: No significant growth
NSTG: National standard treatment guidelines
OFX: Ofloxacin
Cw-PAMS: Commonwealth Partnerships for Antimicrobial Stewardship
PPS: Point prevalence survey
QSE: Quality system essentials
ROX: Roxithromycin
SAM: Ampicillin–sulbactam
SPX: Sparfloxacin
STG: Standard treatment guidelines
SXT: Trimethoprim–sulfamethoxazole
TAT: Turn-around time
TCY: Tetracycline
TDR: Special Programme for Research and Training in Tropical Diseases
TZP: Piperacillin–tazobactam
UTI: Urinary tract infection
VRSA: Vancomycin-resistant *Staphylococcus aureus*
WHO: World Health Organization
